# Stromal derived factor-1 mediates the lung regenerative effects of mesenchymal stem cells in a rodent model of bronchopulmonary dysplasia

**DOI:** 10.1186/s12931-017-0620-z

**Published:** 2017-07-12

**Authors:** Joel Reiter, Shelley Drummond, Ibrahim Sammour, Jian Huang, Victoria Florea, Polliana Dornas, Joshua M. Hare, Claudia O. Rodrigues, Karen C. Young

**Affiliations:** 1 Department of Pediatrics, Hadassah-Hebrew University Medical Center, Jerusalem, Israel; 2 Department of Pediatrics, University of Miami Miller School of Medicine, Miami, FL USA; 3 Department of Medicine/Cardiovascular Division, University of Miami Miller School of Medicine, Miami, FL USA; 4 Department of Molecular and Cellular Pharmacology, University of Miami Miller School of Medicine, Miami, FL USA; 5 Batchelor Children’s Research Institute, University of Miami Miller School of Medicine, Miami, FL USA; 6 The Interdisciplinary Stem Cell Institute, University of Miami Miller School of Medicine, Miami, FL USA

## Abstract

**Background:**

Mesenchymal stem cells (MSCs) attenuate lung injury in experimental models of bronchopulmonary dysplasia (BPD). Stromal derived factor-1 (SDF-1), a chemokine secreted by MSCs, modulates angiogenesis and stem cell recruitment. Here we tested the hypothesis that SDF-1 mediates MSC protective effects in experimental BPD by modulating angiogenesis.

**Methods:**

SDF-1 was knocked down in MSCs using lentiviral vectors carrying anti-SDF-1 short hairpin RNA (MSC-SDF KD). Non-silencing short hairpin RNA was used as control (MSC-NS control). Newborn rats exposed to normoxia or hyperoxia (FiO2 = 0.85) for 3 weeks, were randomly assigned to receive a single intra-tracheal injection (IT) of MSC-NS control or MSC-SDF KD (1 × 10^6^ cells/50 μl) or placebo on postnatal day 7. The degree of alveolarization, lung angiogenesis, inflammation, and pulmonary hypertension (PH) were assessed at postnatal day 21.

**Results:**

Administration of IT MSC-NS control improved lung alveolarization, angiogenesis and inflammation, and attenuated PH in newborn rats with hyperoxia-induced lung injury (HILI). In contrast, knockdown of SDF-1 in MSCs significantly reduced their beneficial effects on alveolarization, angiogenesis, inflammation and PH.

**Conclusions:**

The therapeutic benefits of MSCs in neonatal HILI are in part mediated by SDF-1, through anti-inflammatory and angiogenesis promoting mechanisms. Therapies directly targeting this chemokine may provide a novel strategy for the treatment of BPD.

## Background

Bronchopulmonary dysplasia (BPD) is a chronic multifactorial lung disease that affects 25–35% of extremely low birth weight preterm infants [1]. This disease is characterized by an arrest of alveolar development, decreased angiogenesis, and in the most severe cases, pulmonary vascular remodeling and right ventricular failure [2, 3]. Unfortunately, there are few therapeutic approaches which decrease the incidence of this condition and survivors have an increased risk of neurodevelopmental delay [4].

Recent reports suggest that bone marrow-derived mesenchymal stem cells (MSC) may be a potential strategy to decrease the incidence of BPD [5]. Bone marrow-derived MSCs are a population of stem cells particularly attractive for therapy as they are easy to expand, and have low risk of immune-rejection [6]. In experimental models of BPD, intra-tracheal (IT) as well as intravenous administration of bone marrow-derived MSCs restored alveolar and vascular structures, decreased vascular remodeling, pulmonary hypertension (PH), and right ventricular hypertrophy [6–8]. Engraftment and differentiation rates of MSCs in these studies were however very low. Furthermore, subsequent work revealed that IT administration of MSC or MSC-conditioned medium resulted in similar short-term regenerative effects in experimental models of BPD, implying a paracrine-mediated mechanism of repair [9]. This is a plausible concept as MSCs are known to secrete several anti-inflammatory cytokines and growth factors which affect cell proliferation, differentiation and survival [6, 7]. Although, recent studies have suggested that keratinocyte growth factor and angiopoetin-1 may partially mediate the reparative effect of MSCs in acute lung injury [10] the specific factors secreted by MSCs responsible for lung repair in BPD are currently unknown.

Stromal derived factor-1 (SDF-1), also called chemokine ligand 12 (CXCL-12), is a cytokine known to play a crucial role in development and organ repair after injury [11]. Downstream signaling following binding to its receptors, chemokine receptor 4 (CXCR4) or chemokine receptor 7 (CXCR7) modulates cell adhesion, migration, proliferation, survival, and differentiation [12]. SDF-1 knockout mice show cardiac defects and abnormalities in vasculogenesis and hematopoiesis [13]. Increased local production of SDF-1 following tissue injury is an important factor in the reparative cascade, as this chemokine mediates homing and engraftment of stem cells to injured areas [11]. In an effort to capitalize on the stem cell chemoattractant properties of SDF-1, several investigators have tried to ascertain whether prolongation of SDF-1 effects would promote organ repair. In a pre-clinical study, SDF-1 gene transfer enhanced ischemia-induced vasculogenesis and angiogenesis in vivo [14]. Moreover, in a recent phase 1 open-label dose-escalation study, investigators used plasmid DNA to deliver SDF-1 to the myocardium of patients with ischemic cardiomyopathy, and found that patients receiving the highest doses of SDF-1 had an improvement in their quality of life [15]. Interestingly, bone marrow-derived MSCs secrete SDF-1, and SDF-1/CXCR4 autocrine signaling enhances MSC adhesion, growth, migration, survival and differentiation [16]. Previous studies have also shown that over-expression of SDF-1 by MSCs improves cardiac function, increases neo-vascularization and decreases cardiomyocyte apoptosis in a rodent model of myocardial infarction [17]. Whilst specific knockdown of SDF-1 expression in MSCs reduced the efficiency of these cells to improve wound closure, overexpression improved healing by promoting neovascularization [18]. There are currently no published reports evaluating the role of SDF-1 in MSC based-repair of the injured neonatal lung. Indeed, while some investigators have suggested that SDF-1 and its receptor, CXCR4, augment pulmonary fibrosis [19]; other reports suggest a crucial role of SDF-1 in neo-alveolarization [20].

The present study sought to ascertain whether SDF-1 mediates the reparative effects of IT bone marrow-derived MSCs in an experimental rodent model of BPD. Our results indicate that the therapeutic benefits of MSC administration in experimental BPD are in part mediated by SDF-1, through pro-angiogenic and anti-inflammatory mechanisms. Together, our findings demonstrate that SDF-1 is an essential mediator of MSC reparative effects in experimental BPD, and suggest a role for this chemokine in repair of the neonatal lung.

## Methods

### Animals

Pregnant Sprague Dawley rats were purchased from Charles River Laboratories (Wilmington, MA). Rats and newborn pups were treated according to National Institute of Health guidelines for the use and care of laboratory animals following approval of the study protocol by the University of Miami Animal Care and Use Committee.

### Mesenchymal stem cell retrieval

Rats were euthanized by CO_2_ asphyxiation. Tibiae and femurs were aseptically harvested and cleaned of adherent soft tissue. The proximal and distal ends of the femur were excised at the beginning of marrow cavity. Whole bone marrow plugs were harvested by flushing the bone marrow cavity with a syringe (18-gauge needle) filled with phosphate buffered saline +1% fetal bovine serum. The cells were treated with red cell lysis buffer, washed and re-suspended in alpha minimum essential medium supplemented with 20% fetal bovine serum, 1% penicillin-streptomycin, and 1% L-glutamine. After 3 days of culture, the non-adherent cells were removed and fresh media added. Medium was changed every 3 to 4 days until the adherent MSCs were confluent. MSCs were characterized by flow cytometry by staining with antibodies against CD29, CD45 and CD90, as previously reported [21]. Results were compared to isotype control staining.

### Lentiviral vector preparation and SDF-1 knockdown

Plasmid constructs expressing anti-mouse SDF-1 and non-silencing (NS) short hairpin RNAs were used to prepare lentiviral vectors for SDF-1 knockdown and control. Viral packaging was performed by transfection of human embryonic kidney cells with lipofectamine 2000 (Invitrogen, Carlsbad, CA) as previously described [22]. After collection from culture supernatants, viral particles were precipitated and concentration determined by ELISA using a commercially available kit (Cell Biolabs, San Diego, CA). Rat MSCs were transduced with equal amounts of NS-control and anti-mouse SDF-1 lentiviruses in media supplemented with 2% serum. Transduction efficiency was determined by expression of green fluorescent protein, and positive cells selected with puromycin. Knockdown was confirmed by Western blot analysis for SDF-1. Different cell batches expressing NS-control and anti-SDF-1 short hairpin RNA were frozen for storage. Prior to injections, cells were thawed and cultured for at least 1 week. After harvesting, cell viability was estimated with trypan blue and re-suspended at 1 X 10^6^ cells/50 μl for IT administration.

### Neonatal hyperoxia model

Sprague Dawley pups assigned to normoxia (RA: 21% O_2_) or hyperoxia (85% O_2_) from postnatal day 1 to postnatal day 21 were randomly assigned to receive IT injections of MSC expressing anti-SDF-1 (MSC-SDF KD), non-silencing control short hairpin RNA (MSC-NS control), or phosphate buffered saline as placebo (PL) on postnatal day 7. This route was chosen as prior studies have demonstrated that IT MSCs are more effective than systemically administered cells [23]. Additionally, since all MSCs were re-constituted in phosphate buffered saline, the latter was utilized as placebo. Oxygen exposure was achieved in a Plexiglas chamber by a flow-through system and the oxygen level inside the chamber was monitored daily with a Maxtec oxygen analyzer (Model OM25-RME; Maxtec, Salt Lake City, Utah). Mothers were rotated every 48 h between the hyperoxia and normoxia chambers to prevent damage to their lung. Litter size was adjusted to 10–12 pups to control for the effect of litter size on nutrition and growth. Hemodynamic measurements, lung morphometric and molecular studies were performed on postnatal day 21.

### MSC administration and engraftment

On postnatal day 7, pups assigned to normoxia (21% O_2_) or hyperoxia (85% O_2_) were anesthetized with intra-peritoneal ketamine (30 mg/kg) and xylazine (4 mg/kg), and the trachea was exposed through a small incision in the midline of the neck. MSC (1 × 10^6^ in 50 μl PBS) expressing NS-Control or anti-SDF-1 short hairpin RNA were delivered by tracheal puncture with a 30-gauge needle. PBS was injected as placebo. The incision was closed with VetbondTM tissue adhesive (Santa Cruz Biotechnology, Dallas, Texas) and the pups were allowed to recover within a warmed plastic chamber, and when vigorous, returned to their mother.

### Lung morphometric analysis

Lung morphometric analysis was performed as previously described [24, 25]. Briefly, the trachea and pulmonary artery were perfused and fixed overnight in 4% paraformaldehyde, and embedded in paraffin. Serial sections 5 μm thick were taken from the upper and lower lobes, and stained with hematoxylin and eosin. Images from 5 randomly selected, non-overlapping parenchymal fields were acquired from lung sections of each animal (5–6/group) using an Olympus Qcolor 3 color camera interfaced with a light microscope (Model Leica DMI 4000B) at 20X magnification. Alveolarization was determined by calculating the mean linear intercept (MLI) and alveolar volume fraction. Mean linear intercept (MLI) was calculated by determining average distance between intersects of alveolar septal tissue and a superimposed counting grid. Alveolar volume fraction within the parenchyma was assessed by point counting. Grid intersections were treated as points (81 per slide), 5–6 slides per group. The number of points in alveoli (excluding respiratory bronchioles and alveolar ducts) was divided by the number of points in the parenchyma (excluding large vessels and bronchi) as previously described [26].

### Pulmonary vascular density

Lung sections were de-paraffinized, rehydrated, and stained with polyclonal rabbit anti-human Von Willebrand Factor (vWF; Dako Corp, Carpintaria, CA). The number of vessels (20–50 μm diameters) per high power field (HPF) was quantified in 5 randomly selected, non-overlapping, parenchymal fields from lung sections of each animal (*n* = 5–6/group). The concentration of vascular endothelial growth factor (VEGF) in lung homogenates was quantified in parallel using an ELISA kit (R&D Systems; Minneapolis, MN) according to manufacturer specifications.

### Pulmonary vascular remodeling

Paraffin embedded sections were stained with polyclonal rabbit anti-human vWF and monoclonal mouse anti-α-smooth muscle actin (α-SMA: 1:500, Sigma-Aldrich; St. Louis, MO). Medial wall thickness (MWT) of partially and fully muscular arteries (20–50 μm) was determined by using the formula: 2(MT) × 100/ED, where MT is the distance between the internal and external boundaries of the α-SMA layer and ED is the external diameter. Approximately 20 randomly chosen arteries were evaluated per slide (*n* = 5–6/group), and all morphometric analyses were performed by a blinded observer.

### Hemodynamic studies

Pups were anesthetized and right ventricular systolic pressure (RVSP) evaluated as previously described [25]. Briefly, a thoracotomy was performed and a 22 gauge needle connected to a pressure transducer was inserted into the right ventricle. RVSP was measured and recorded on a Gould polygraph (Model TA-400, Gould instruments). Right ventricular hypertrophy was determined by measuring the weight ratio of the right ventricle (RV) to the left ventricle (LV) and septum (S).

### Lung inflammation

Broncho-alveolar lavage fluid analysis was performed as previously described [27]. Briefly, a 20 gauge angiocatheter was inserted into the trachea and secured in place with a 4.0 silk suture. The lungs were lavaged by infusing and then aspirating four aliquots of normal saline (0.5 ml each) into the lungs. The broncho-alveolar lavage fluid obtained was then centrifuged for 5 min. The cells were washed with normal saline and quantified using a hemocytometer. In addition, cells were suspended in 1000 μl of normal saline and affixed to slides using an Eppendorf centrifuge. Differential cell counts were performed on the cytospin preparations after Giemsa staining. Lung inflammation was also assessed by immunostaining for MAC-3 (BD Biosciences, San Jose, CA), a macrophage-specific marker. The number of MAC-3-positive cells in the alveolar air spaces was counted from 10 random images taken with the 20X objective on each slide, (*n* = 5–6/group). Lung monocyte chemoattractant protein (MCP-1) and interleukin-10 (IL-10) concentration were determined by ELISA as per manufacturer specifications (Abcam, Cambridge, MA).

### Western blot analysis

The protein expression of Interleukin-1β (IL-1β) and SDF-1 in lung homogenates was determined by Western blot analysis. The monoclonal antibody for IL-1β (1:1000) and the polyclonal antibody for SDF-1 (1:50) were obtained from Cell Signalling Technology (Danvers, MA). Lung homogenates were separated by 10% SDS-PAGE, transferred to nitrocellulose membranes, and blocked overnight at 4 °C in 5% bovine serum albumin. Immunodetection was performed by incubating the membranes with the primary antibodies diluted in blocking buffer for 1 h at room temperature. After washing, a semilumiscent horseradish peroxidase substrate was diluted in blocking buffer and applied for 60 min. Band intensity was quantified with Quantity One software (Bio-Rad, Hercules, CA), with β-Actin acting as the normalization protein (1:10,000; Sigma-Aldrich, St. Louis, MO).

### Statistical analysis

Results, reported as mean ± standard deviation (SD), were analyzed by two-way ANOVA with post-hoc Holm-Sidak test using SigmaStat software. *P* values less than 0.05 were considered statistically significant.

## Results

### SDF-1 knockdown in MSC

**Fig. 1:**
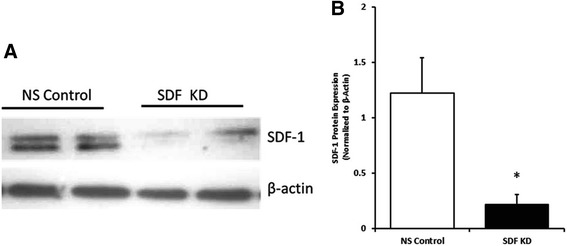
Knockdown of SDF-1 in Rat Mesenchymal Stem Cells (MSCs). **a** Representative Western blot image of non-silencing control (NS Control) and SDF-1 knockdown (SDF KD) samples of independent lentiviral transductions. **b** Quantification of SDF-1 knockdown by densitometry analysis. Results were normalized to β-actin expression.*p < 0.05, *n* = 3/group

Rat MSCs isolated by adherence to plastic were subjected to flow cytometry analysis for characterization. 95.7% and 97.8% of the cell population were positive for CD29 and CD90, respectively, while only 0.2% was positive for CD45. In order to investigate the role of SDF-1 in MSC regenerative potential in neonatal hyperoxia-induced lung injury (HILI), rat MSCs were transduced with lentiviral vectors expressing short hairpin RNA against SDF-1 or non-silencing short hairpin RNA as control. Western blot analysis revealed that the degree of SDF-1 knockdown achieved in MSC was 80% relative to control (Fig. 1a and b).

### SDF-1 knockdown attenuates MSC regenerative effects on lung alveolarization

**Fig. 2:**
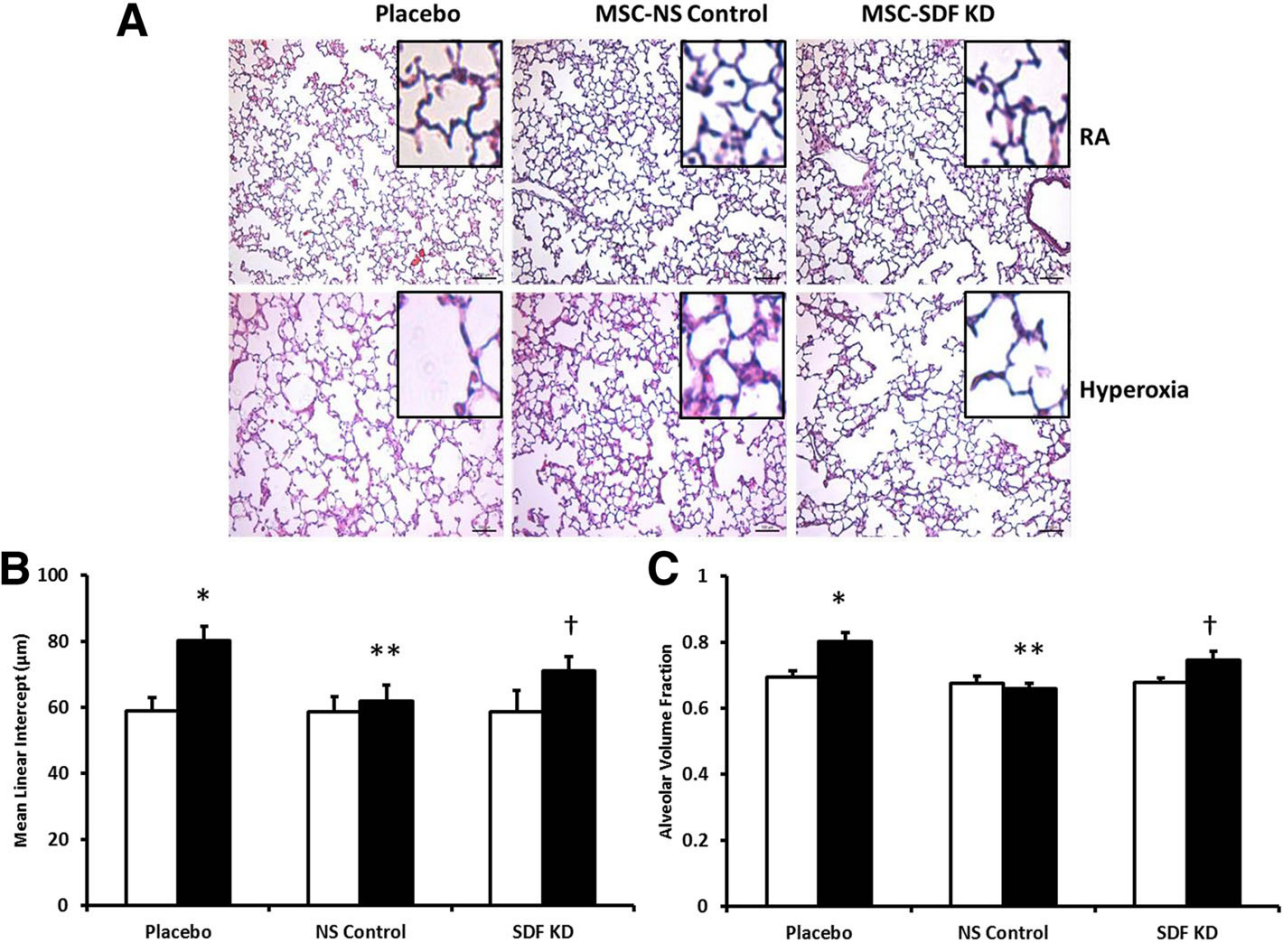
SDF-1 Knockdown Attenuates MSC Regenerative Potential On Lung Alveolarization. **a** Hematoxylin and eosin stained lung sections demonstrating improved alveolar structure in hyperoxia-exposed pups treated with IT MSC-NS control. This improvement was significantly decreased following SDF-1 knockdown in MSC (MSC-SDF KD). Original magnification X 200. Scale bars are 100 μm. Morphometric analyses revealed that while IT MSC-NS control reduced the (**b**) mean linear intercept and (**c**) alveolar volume fraction in hyperoxic animals, administration of IT MSC SDF KD reversed these beneficial effects. (*P < 0.05; normoxia vs. hyperoxia-placebo, ** *P* < 0.05; hyperoxia-placebo vs hyperoxia MSC-NS control, † *P* < 0.05; hyperoxia MSC-NS control vs. hyperoxia MSC-SDF KD; *N* = 5–7/group). White bars are room air (RA) and black bars are hyperoxia

Similar to BPD, exposure of neonatal pups to hyperoxia decreased lung alveolarization as evidenced by larger, simplified alveoli with fewer secondary crests (Fig. 2a). IT administration of MSC-NS control improved alveolar structure in hyperoxic animals, but this regenerative effect was attenuated in the MSC-SDF KD treated group (Fig. 2a). Morphometric analysis revealed an increase in MLI (59 ± 4 vs. 81 ± 4 μm; RA-PL vs. hyperoxia-PL, *p* < 0.001; *n* = 5–7/group), as well as alveolar volume fraction in hyperoxia exposed rats (0.69 ± 0.02 vs. 0.8 ± 0.03; RA-PL vs. hyperoxia-PL, *p* < 0.001; *n* = 5–7/group). In contrast, IT MSC-NS control significantly decreased MLI (81 ± 4 vs. 62 ± 5 μm; hyperoxia-PL vs. hyperoxia MSC-NS control; *p* < 0.001; *n* = 5–7/group) and alveolar volume fraction (Fig. 2b and c). SDF-1 knockdown in MSC attenuated this improvement in MLI (71 ± 4 vs. 62 ± 5 μm; hyperoxia MSC-SDF KD vs. hyperoxia MSC-NS control; *p* < 0.004; *n* = 5–7/group) and alveolar volume fraction (Fig. 2b and c), suggesting that MSC beneficial effects on alveolarization are mediated by SDF-1.

### Pro-angiogenic effects of MSCs in experimental BPD are mediated by SDF-1

**Fig. 3:**
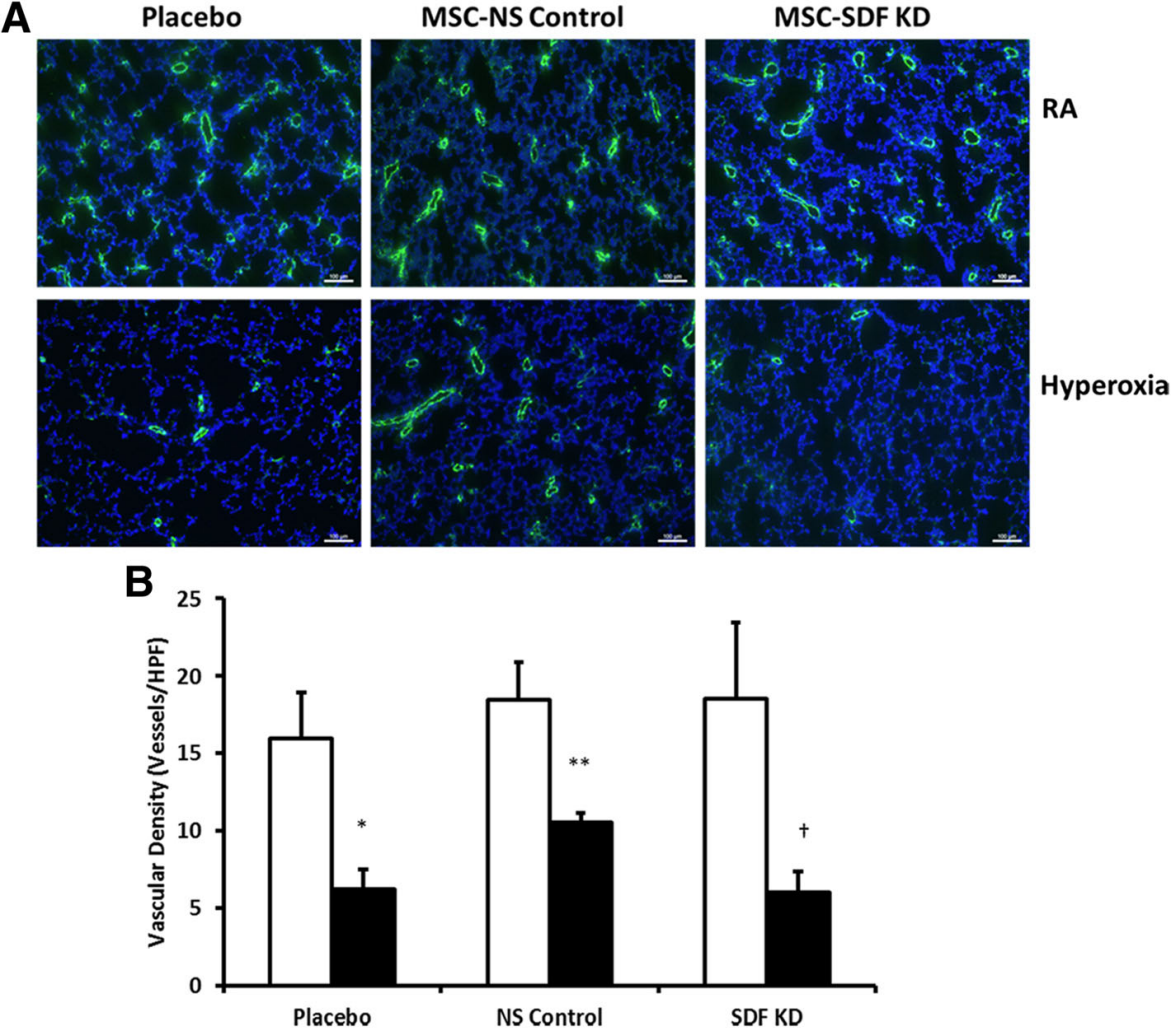
SDF-1 Knockdown Attenuates MSC Regenerative Potential On Lung Angiogenesis. **a** Lung sections stained with Von Willebrand Factor (green) and 4′6-diamidino-2-phenylindole (DAPI: blue), demonstrating improved vascular density in hyperoxia-exposed pups treated with IT MSC-NS control. SDF-1 knockdown obliterated the beneficial effects of MSC on angiogenesis in experimental BPD. Original magnification X 100. Scale bars are 100 μm. **b** IT MSC-NS control increased vascular density in hyperoxic animals but administration of IT MSC-SDF KD reversed these beneficial effects. (*P < 0.05; normoxia vs. hyperoxia-placebo, ** *P* < 0.05; hyperoxia-placebo vs hyperoxia MSC-NS control, † *P* < 0.05; hyperoxia MSC-NS control vs. hyperoxia MSC-SDF KD; *N* = 5–7/group). White bars are room air (RA) and black bars are hyperoxia

There was no difference in vascular density between the RA groups. Exposure of neonatal pups to hyperoxia reduced vascular density (Fig. 3a and b) as evidenced by decreased number of vessels per HPF (16 ± 3 vs. 6 ± 1 vessels/HPF; RA-PL vs. hyperoxia-PL, *p* < 0.001; *n* = 6/group). IT administration of MSC-NS control improved lung angiogenesis (6 ± 1 vs. 11 ± 0.6 vessels/HPF; hyperoxia-PL vs. hyperoxia MSC-NS control; *p* < 0.02; *n* = 5–6/group). Knockdown of SDF-1 in MSC markedly attenuated the angiogenic effects of MSCs in HILI (6 ± 1 vs. 11 ± 1 vessels/HPF; hyperoxia MSC-SDF KD vs. hyperoxia MSC-NS control; *p* < 0.002; *n* = 5/group). There was no difference in lung VEGF concentration between the hyperoxia MSC treated groups (750 ± 57 vs. 750 ± 16 pg/ml; hyperoxia MSC-SDF KD vs. hyperoxia MSC-NS control; *n* = 5/group).

### SDF-1 knockdown attenuates MSCs beneficial effects on neonatal hyperoxia-induced PH

**Fig. 4:**
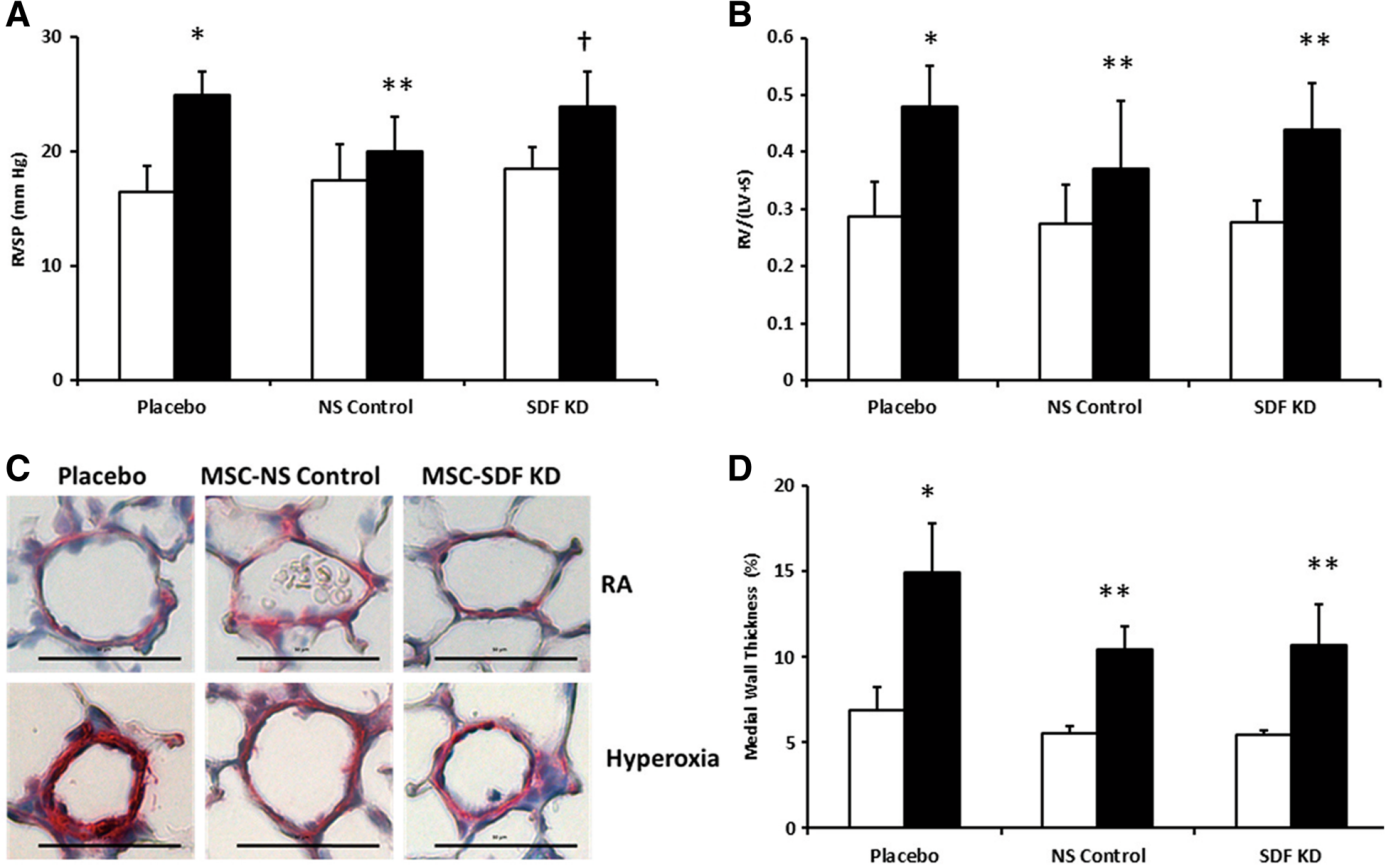
Effects of SDF-1 Knockdown in MSC on Pulmonary Hypertension (PH) and Vascular Remodeling in Experimental BPD. **a** IT MSC-NS control significantly decreased right ventricular systolic pressure (RVSP) in hyperoxic animals. SDF-1 knockdown reduced the beneficial effects of MSC on PH. (*P < 0.05; normoxia vs. hyperoxia-placebo, ** *P* < 0.05; hyperoxia-placebo vs hyperoxia MSC-NS control, † *P* < 0.05; hyperoxia MSC-NS control vs. hyperoxia MSC-SDF KD; *N* = 9–20/group). **b** No significant differences in the RV/LV + S (weight ratio of right ventricle to left ventricle and septum) between the MSC treated groups (*P < 0.05; normoxia vs. hyperoxia-placebo, ** *P* < 0.05; hyperoxia-placebo vs hyperoxia MSC-NS control or hyperoxia MSC-SDF KD; *N* = 15–20/group). **c** Lung sections stained with α-smooth muscle actin (red) demonstrating decreased vascular remodeling in hyperoxia-exposed pups treated with IT MSC-NS control or IT MSC-SDF KD. Original magnification X 400. Scale bars are 50 μm. **d** No significant differences in the medial wall thickness (%) between the MSC-NS control and MSC-SDF KD treated groups (*P < 0.05; normoxia vs. hyperoxia-placebo, ** *P* < 0.05; hyperoxia-placebo vs hyperoxia MSC-NS control or hyperoxia MSC-SDF KD; *N* = 5–7/group).White bars are RA and black bars are hyperoxia

Hyperoxia-exposed neonatal pups developed significant PH (Fig. 4a and b) as evidenced by increased RVSP (14 ± 3 vs. 25 ± 2 mmHg; RA-PL vs. hyperoxia-PL, *p* < 0.001; *n* = 15–20/group) and RV/LV + S (0.28 ± 0.06 vs. 0.58 ± 0.17; RA-PL vs. hyperoxia-PL, *p* < 0.001; *n* = 15–20/group). IT administration of MSC-NS control significantly improved both of these measures of PH (Fig. 4a and b). In contrast, knock down of SDF-1, attenuated these beneficial effects of MSCs on RVSP (24 ± 3 vs. 20 ± 3 mmHg; hyperoxia MSC-SDF KD vs. hyperoxia MSC-NS control; *p* < 0.004; *n* = 9–15/group). There was no significant difference in the RV/LV + S (0.44 ± 0.07 vs. 0.37 ± 0.12; hyperoxia MSC-SDF KD vs. hyperoxia MSC-NS control; *n* = 15–20/group) or pulmonary vascular remodeling between the hyperoxia MSC treated groups (Fig. 4b-d).

### SDF-1 knockdown attenuates the beneficial effects of MSC on neonatal hyperoxia-induced lung inflammation

**Fig. 5:**
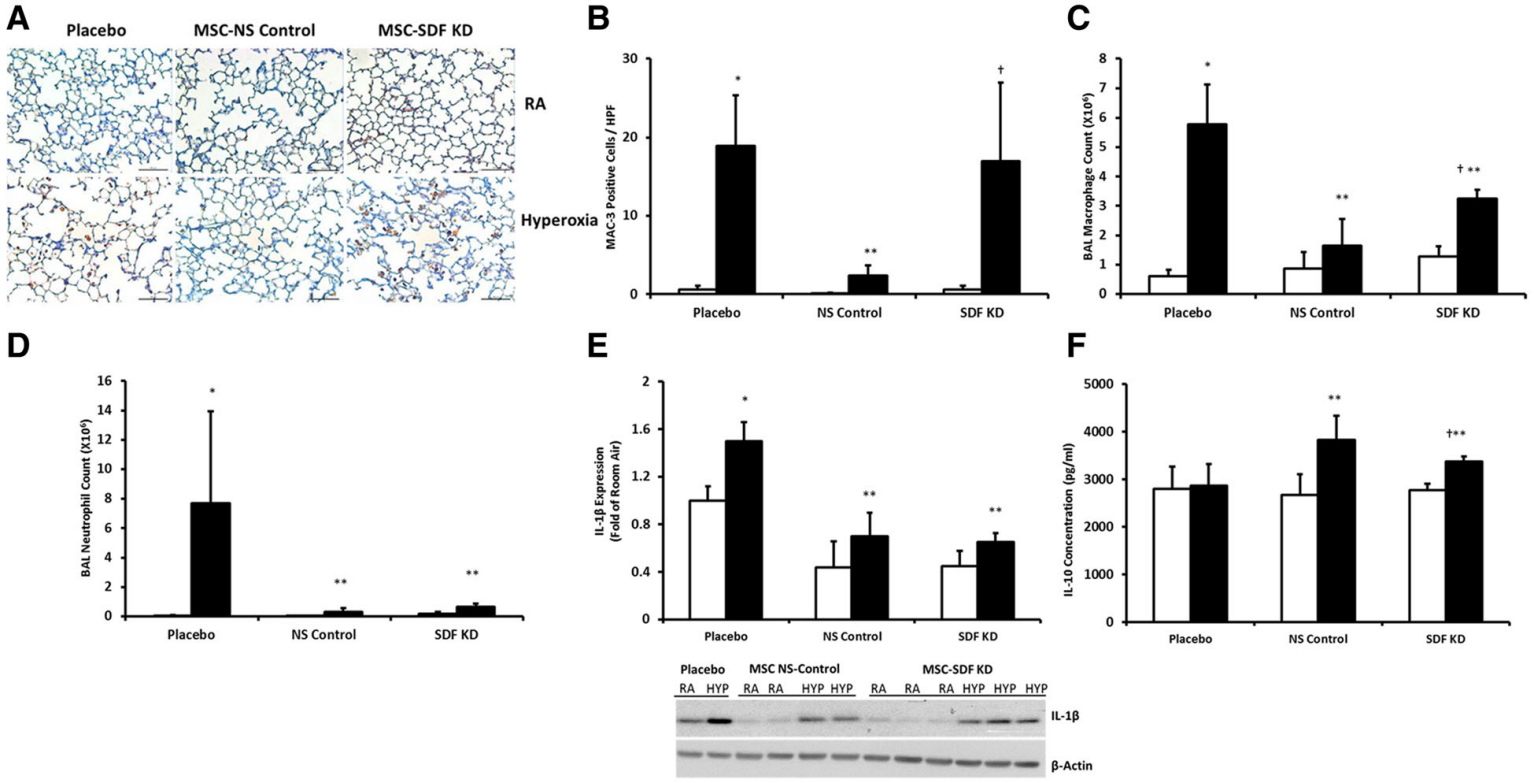
Effect of SDF-1 Knockdown on MSC Anti-inflammatory Effects in Experimental BPD. **a** Lung sections stained with MAC-3 (brown) showing increased MAC-3 positive cells/HPF in hyperoxic animals treated with IT MSC-SDF KD. Original magnification X 200. Scale bars are 100 μm. **b** IT MSC-NS control significantly reduced MAC-3 positive cells/HPF and (**c**) broncho-alveolar lavage fluid macrophage count in hyperoxic animals. SDF-1 knockdown decreased this beneficial effect of MSC. **d** No differences in broncho-alveolar lavage fluid neutrophil count between the hyperoxic MSC treated groups. **e** Similar decrease in lung IL-1β expression in hyperoxia MSC-NS control and MSC-SDF KD groups. A representative Western Blot normalized to β-Actin is showed in the lower panel. **f** Increased lung Il-10 concentration in both hyperoxic MSC treated groups but this is blunted in the hyperoxia MSC-SDF KD group. (*P < 0.05; normoxia vs. hyperoxia-placebo, ** *P* < 0.05; hyperoxia-placebo vs hyperoxia MSC-NS control or hyperoxia MSC-SDF KD, † *P* < 0.05; hyperoxia MSC-NS control vs. hyperoxia MSC-SDF KD; *N* = 5/group). White bars are RA and black bars are hyperoxia. HYP is hyperoxia

Exposure of neonatal pups to hyperoxia for 3 weeks triggered substantial lung inflammation, characterized by increased MAC-3 immunostaining and broncho-alveolar lavage fluid inflammatory cell count (Fig. 5a-d). Quantification revealed a significant increase in the number of MAC-3 positive cells/HPF (Fig. 5b) in hyperoxia-exposed lungs (0.6 ± 0.5 vs. 19 ± 6 cells/HPF; RA-PL vs. hyperoxia-PL, *p* < 0.001; *n* = 5/group). Broncho-alveolar lavage fluid analysis also showed an increase in the number of macrophages (0.6 ± 0.2 vs. 3.4 ± 2.8 × 10^6^ cells/ml; RA-PL vs. hyperoxia-PL, *p* < 0.001; *n* = 5/group) and neutrophils (Fig. 5c and d). Administration of IT MSC-NS control significantly decreased MAC-3 positive cell counts/HPF (19 ± 6 vs. 2 ± 1 cells/HPF; hyperoxia-PL vs. hyperoxia MSC-NS control, *p* < 0.001; *n* = 5/group), and broncho-alveolar lavage fluid inflammatory cell count (Fig. 5a-d).

The anti-inflammatory effect of MSCs was significantly reduced by SDF-1 knockdown (Fig. 5a-d). IT administration of MSC-SDF KD did not decrease the number of MAC-3 positive cell counts/HPF in hyperoxic lungs (16 ± 10 vs. 2 ± 1 cells /HPF; hyperoxia MSC-SDF KD vs. hyperoxia MSC-NS control, *p* < 0.001; *n* = 5/group), Fig. 5b. Broncho-alveolar lavage fluid analysis also revealed that IT MSC-SDF KD attenuated the reduction in the number of broncho-alveolar lavage fluid macrophages promoted by MSC (3.3 ± 0.3 vs. 1.6 ± 0.9 × 10^6^; hyperoxia MSC-SDF KD vs. hyperoxia MSC-NS control, *p* < 0.001; *n* = 5/group), Fig. 5c. There was no significant difference in broncho-alveolar lavage fluid neutrophil count between hyperoxia-exposed MSC treated groups (Fig. 5d). In addition, while administration of MSC-NS control and MSC-SDF KD to hyperoxic pups significantly decreased lung MCP-1 concentration (data not shown) and interleukin-1β expression (Fig. 5e), there was no difference in the expression or concentration of these cytokines between the hyperoxia MSC treatment groups. Interestingly, as compared to the hyperoxia-PL treated group, there was however a significant increase in the concentration of the anti-inflammatory cytokine, IL-10 in the hyperoxia-exposed MSC-NS control and MSC-SDF KD treated pups, but this increase was blunted in the hyperoxia-exposed MSC-SDF KD treated pups (Fig. 5f).

## Discussion

Despite advances in neonatal care over the past 2 decades, BPD remains a significant cause of morbidity and mortality in neonates. Recent literature from the National Institute of Child Health reviewing trends in neonatal care and outcomes among extremely low birth weight infants (ELBWs) showed a significant improvement in the survival to discharge of preterm ELBWs, but this was accompanied by an increase in BPD rates [28]. While this increase in BPD rates could be secondary to the improved survival of ELBWs, therapies for BPD have remained elusive and survivors have significantly higher rates of motor, cognitive and behavioral impairment [29].

Interestingly, emerging evidence now suggests that IT administration of MSCs may regenerate the injured preterm lung. Pre-clinical studies, including those performed in our laboratory, demonstrate that IT MSCs improve alveolar and vascular development, reverse PH and improve pulmonary vascular remodeling in experimental models of BPD [8, 9]. In a seminal study by van Haaften and colleagues, IT delivery of MSCs not only restored alveolar and vascular structure in experimental BPD but also improved survival and exercise tolerance though engraftment was minimal [8]. In a similar study by Aslam et al., intravenous administration of MSCs improved alveolarization and decreased PH in neonatal mice with experimental BPD but the administration of MSC-conditioned medium had a more pronounced reparative effect, suggesting that the effect was mainly paracrine mediated [30]. In fact, several recent studies utilizing MSCs to regenerate the injured lung have shown that whilst MSCs do indeed have the ability to conform to a specific identity in response to local host factors, the engraftment and differentiation of MSCs into lung epithelial cells in vivo is very uncommon [8, 30, 31].

The exact mechanisms by which MSCs alleviate BPD are unclear. Multiple reports have demonstrated that MSCs have immunomodulatory, pro-angiogenic, anti-oxidant and anti-apoptotic effects in experimental models of BPD [8, 9, 30]. In addition, lung MSCs are decreased in neonatal rodents with experimental BPD [8] and potentially exogenous MSCs could re-establish a regenerative environment and protect this endogenous MSC pool. In this manuscript, we provide new evidence that SDF-1 in part mediates the regenerative effects of MSCs in neonatal HILI, an experimental model of BPD. We show that knockdown of SDF-1 in MSCs attenuates their alveolar and vascular protective effects. Most striking were the effects on angiogenesis as well as the suppression of MSC anti-inflammatory potential following SDF-1 knockdown. These findings not only demonstrate that SDF-1 is a crucial mediator of MSC reparative effects in experimental BPD, but importantly suggests a potential role for this chemokine in repair of the injured preterm lung.

SDF-1 plays a pivotal role in stem cell migration, adhesion, proliferation, survival and differentiation. Expressed in multiple cells within the lung, including endothelial and epithelial cells [32], the role of this chemokine in lung development and repair remains controversial. SDF-1 knockout mice have emphysematous changes in the lung architecture [13] and platelet derived SDF-1 primes pulmonary capillary endothelial cells to initiate regeneration of the lung following pneumonectomy [20]. SDF-1 expression is decreased in the pleural fluid of patients with prolonged air leak and exposure of alveolar epithelial cells to SDF-1 enhances alveolar cell migration and promotes wound scratch closure [33]. Neonatal rodents with experimental BPD have unchanged lung SDF-1 expression [34], while lung tissue of patients with idiopathic pulmonary fibrosis have an increased number of SDF-1 expressing cells and blockade of the SDF-1 receptor, CXCR4, decreases pulmonary fibrosis [19]. Intriguingly, these discrepancies may actually be secondary to the differential signaling of SDF-1. Indeed, after acute injury, selective activation of SDF-1/CXCR7 signaling pathways promotes liver and lung regeneration, however following chronic injury SDF-1/CXCR4 maladaptive signaling pathways dominate, provoking fibrosis [35, 36].

Whether SDF-1 mediates MSC regenerative effects in BPD was heretofore unknown. Prior studies have revealed that over-expression of SDF-1 by MSCs improves cardiac function and decreases cardiomyocyte apoptosis in rodents with myocardial infarction [17]. Conversely, knockdown of SDF-1 in MSCs drastically reduces MSC efficiency in improving wound closure, indicating that SDF-1 secretion by MSCs is largely responsible for MSC’s beneficial action in wound healing [18]. In this study, we provide robust evidence demonstrating that MSC pro-angiogenic effects in experimental BPD are largely secondary to SDF-1 secretion. We show that knockdown of SDF-1 in MSCs obliterates the therapeutic effects of MSCs on lung angiogenesis in hyperoxic pups. This is consistent with other studies which show that SDF-1 recruits angiogenic precursors to sites of injury and acts as a crucial retention signal [37]. Interestingly, although induction of VEGF was shown to be dependent on SDF-1 in ischemic limbs [38], and VEGF knockdown decreased the efficacy of MSCs in neonatal HILI [39], in our present study, there was no difference in VEGF expression in MSCs which were transduced with lentiviral vectors expressing shRNA against SDF-1 as compared to those which received non-silencing shRNA. Moreover VEGF expression was similar in both hyperoxia-treated groups.

Inflammation plays a major role in the pathogenesis of BPD [40]. In our study, neonatal pups with HILI had a significant increase in lung macrophage infiltration and this was decreased following administration of MSC-NS control. This finding is in keeping with other studies demonstrating that MSCs improve neonatal HILI by decreasing inflammation [30]. Surprisingly, SDF-1 knockdown in MSCs significantly suppressed some of MSC anti-inflammatory effects in this model. Indeed, other investigators have demonstrated that SDF-1 participates in lung injury by increasing the migration of pro-inflammatory cells to areas of injury [19]. Our findings are however in agreement with recent evidence that SDF-1 controls monocyte recruitment to sites of inflammation and also modulates monocyte differentiation towards a more pro-angiogenic and anti-inflammatory phenotype by down-regulating the transcription factor, RUNX3 [41]. Moreover, SDF-1 regulates IL-10 secretion in T cells by activation of the mitogen-activated protein kinase cascades (MEK-1/ERK) signaling pathway [42].

Anti-inflammatory, anti-remodeling and pro-angiogenic properties are key mechanisms by which MSCs improve PH in neonatal HILI [9]. In our present study, MSC-NS control significantly attenuated RVSP and this was accompanied by decreased inflammation, reduced pulmonary vascular remodeling, and increased lung vascular density. Interestingly, although SDF-1 knockdown markedly reduced the beneficial effects of MSC on PH, this was most likely due to the pro-angiogenic effects of SDF-1 as there was no difference in the degree of vascular remodeling between the hyperoxia MSC treated groups. Moreover, our findings also suggest that other crucial MSC factors, such as VEGF [43], levels of which were unaffected by SDF-1 knockdown may be involved in the suppression of pulmonary vascular remodeling in HILI.

## Conclusion

The present study provides new evidence that SDF-1 plays a crucial role in the lung protective effects of MSCs in experimental BPD. We show that knock down of SDF-1 in MSCs, significantly diminishes the improvement seen in alveolarization, lung vascular density, inflammation and PH following administration of IT MSC. These findings add to the growing body of evidence that strategies which modulate SDF-1 secretion in MSCs may augment the lung regenerative effects of these cells in BPD. Moreover, although further investigation will be needed, this study also suggests that therapies that augment lung SDF-1 expression may be potentially efficacious for the treatment of BPD.

## Abbreviations

BPD: Bronchopulmonary dysplasia
CXCL-12: Chemokine ligand 12 (SDF-1)
CXCR4: Chemokine receptor 4
CXCR7: Chemokine receptor 7
HILI: Hyperoxia induced lung injury
HPF: High power field
IT: Intra-tracheal
MLI: Mean linear intercept
MSC-NS Control: SDF-1 non-silenced mesenchymal stem cell control (sham knockdown)
MSCs: Mesenchymal stem cells
MSC-SDF KD: SDF-1 knocked down mesenchymal stem cells
MWT: Medial wall thickness
PH: Pulmonary hypertension
RA: Room air, normoxia
RV/LV + S: Weight ratio of the right ventricle to left ventricle and septum
RVSP: Right ventricular systolic pressure
SDF-1: Stromal derived factor-1
VEGF: Vascular endothelial growth factor
α-SMA: Anti-α-smooth muscle actin
